# The complete chloroplast genome sequence of *actinidia valvata*

**DOI:** 10.1080/23802359.2020.1764402

**Published:** 2020-05-13

**Authors:** Yi-ting Chen, Rui-lian Lai, Chun-zhen Cheng, Min-xia Gao, Wen-guang Chen, Ru-jian Wu, Xin Feng

**Affiliations:** a Fruit Research Institute, Fujian Academy of Agricultural Sciences, Fuzhou, P. R. China; b College of Horticulture, Fujian Agriculture and Forestry University, Fuzhou, P. R. China

**Keywords:** *Actinidia valvata*, chloroplast genome, phylogenetic analysis

## Abstract

In this study, we first presented the complete chloroplast genome of *Actinidia valvata* by using Illumina Novaseq sequencing. Its complete chloroplast genome is 156,596 bp in length, containing a large single copy region of 88,477 bp and a small single copy region of 20,379 bp separated by a pair of inverted repeat regions of 23,870 bp. The chloroplast genome contains 112 unique genes, including 78 protein-coding genes, 30 tRNA, and four rRNA genes. Phylogenetic analysis based on chloroplast genome sequences of ten plants from the family Actinidiaceae showed that *A. valvata* is more closely related to *A. polygama* than other members.

Kiwifruit is considered to be one of the most successful artificial domesticated fruit trees in the 21st century (Xu et al. 2017). Its fruit is rich in vitamin C, organic acid, amino acid, polysaccharide, folic acid, mineral elements, and many other nutrients. Nowadays, kiwifruit is mainly planted in China, New Zealand, Italy, Greece, France, etc. China is recognized as the origin of kiwifruit and is rich of kiwifruit germplasm resources. According to the latest botanical classification, kiwifruit germplasm resources were divided into 54 species and 21 varieties (Li et al. 2007). The chloroplast genomes of *A. arguta*, *A. kolomikta*, *A. tetramera*, *A. polygama*, *A. callosa*, *A. eriantha*, *A. chinensis*, *A. deliciosa*, and *A. rufa* have been reported (Hudson and Gardner 1988; Yao et al. 2015; Chul Kim et al. 2018; Lan et al. 2018; Chen et al. 2019). *Actinidia valvata*, commonly used as kiwifruit rootstock, is of great stress resistance especially to waterlogging. The exploration of complete chloroplast genome of it can provide insight and evidence for its genetic background and evolution.

The *A. valvata* leaf samples used in this study were collected from Fujian Kiwifruit Germplasm Repository, which was located in Xiqin Town, Yanping District, Nanping City (CHN, 26°31′19.49″N, 118°07′29.38″E). High-quality genomic DNA was extracted using modified CTAB method and was stored at the Fruit Research Institute, Fujian Academy of Agricultural Sciences (No. FJK-Av03). Genomic DNA sequencing was performed using Illumina Novaseq sequencing platform (Personalgene, Nanjing, CHN) to obtain bout 3.67 G bp of high-quality reads of *A. valvata*. The obtained clean reads were used to align with complete chloroplast genome sequences of *A. arguta, A. kolomikta, A. tetramera, A. polygama, A. callosa, A. eriantha, A. chinensis,* and *A. deliciosa* of *Actinidia* and were used for the assembly of its chloroplast genome using CLC Genomics Workbench V8.0 (CLC Bio, Aarhus, Denmark). The assembled chloroplast genome was annotated by using DOGMA (Wyman et al. 2004) and Geneious (Kearse et al. 2012). The annotated complete chloroplast genome of *A. valvata* was deposited in Genbank (Accession number MT353756).

The complete chloroplast genome of *A. valvata* is 156,596 bp in length, containing a pair of inverted repeat (IR) regions of 23,870 bp, a large single copy region of 88,477 bp, and a small single copy region of 20,379 bp. The chloroplast genome of *A. valvata* contains 112 unique genes, including 78 protein-coding genes, 30 tRNA genes, and four rRNA genes. Among these genes, 94 of them exist as single copy, but four protein-coding genes (i.e. *ndhB*, *rps7, ycf15,* and *ycf2*), 10 tRNA genes (i.e. *trnA*-*UGC*, *trnfM-CAU, trnH-GUG, trnl-CAU, trnl-GAU, trnL-CAA, trnM-CAU, trnN-GUU, trnR-ACG,* and *trnV-GAC*), and four rRNA genes (i.e. *4*.*5S*, *5S*, *16S* and *23S* rRNA) present in double copies. In general, the nucleotide composition of complete chloroplast genome of *A. valvata* is 30.88% A, 18.25% G, 18.99% C, and 31.88% T, and the total GC content is 37.24%.

The complete chloroplast genomes of *A. valvata*, *A. arguta*, *A. kolomikta*, *A. tetramera*, *A. polygama*, *A. callosa*, *A. eriantha*, *A. chinensis*, *A. deliciosa*, and *Saurauia tristyla* (as outgroup) of Actinidiaceae were analyzed using MEGA7.0 (with 1,000 bootstrap replicates) (Kumar et al. 2016). Results showed that the chloroplast genomes of members in *Actinidia* were similar to each other, and *A. valvata* is more closely related to *A. polygama* than other members of Actinidiaceae (Figure 1). These results would provide important references for further heredity and evolution researches of *Actinidia*.

**Figure 1.**
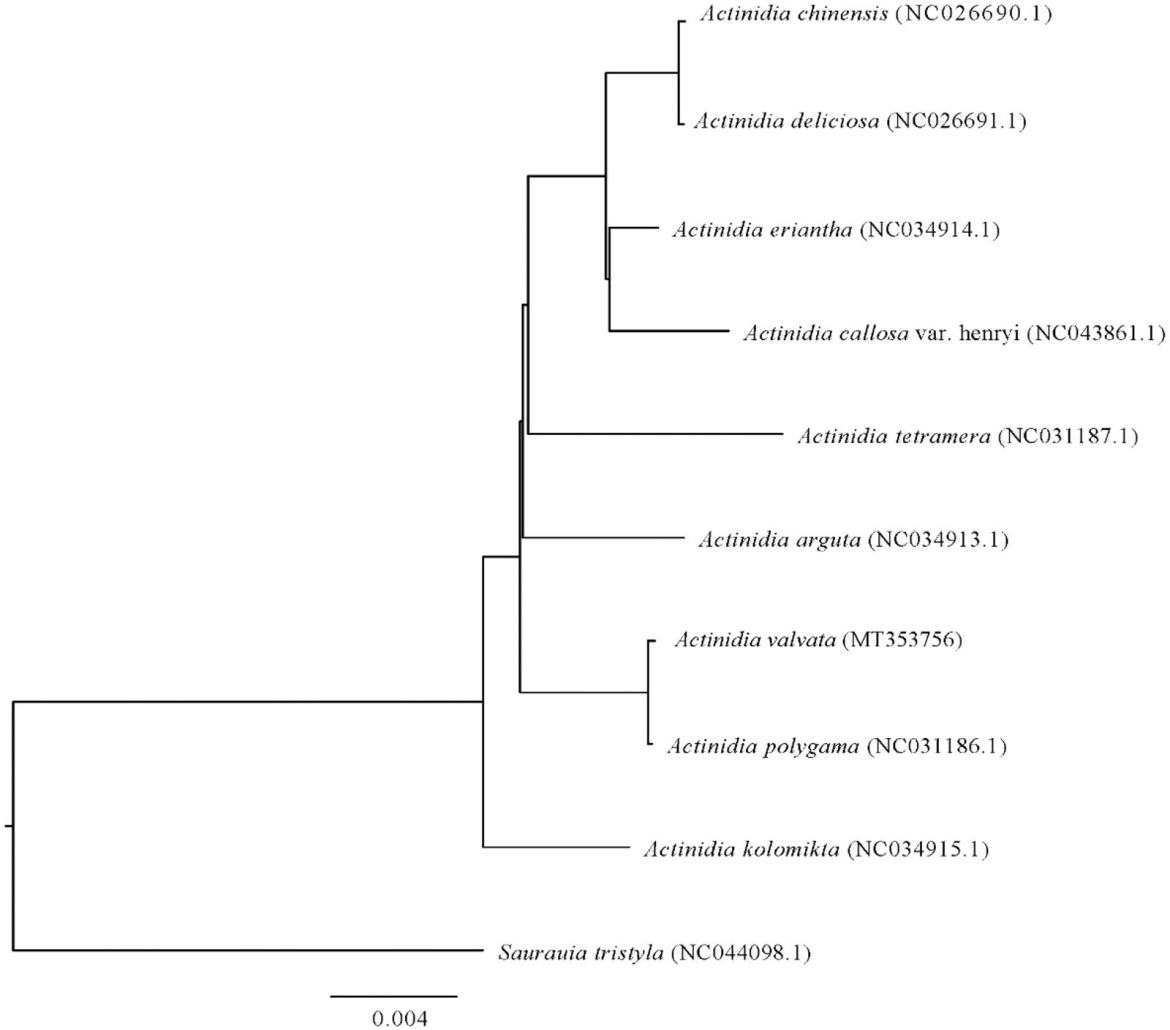
Phylogenetic tree of the complete chloroplast genome sequences from *A. valvata* and other members of Actinidiaceae.

## Data Availability

The data that support the findings of this study is openly available in Genbank at [https://www.ncbi.nlm.nih.gov/genbank/], reference number MT353756.
